# Tumor development in Japanese patients with Lynch syndrome

**DOI:** 10.1371/journal.pone.0195572

**Published:** 2018-04-19

**Authors:** Chiaki Saita, Tatsuro Yamaguchi, Shin-ichiro Horiguchi, Rin Yamada, Misato Takao, Takeru Iijima, Rika Wakaume, Tomoyuki Aruga, Taku Tabata, Koichi Koizumi

**Affiliations:** 1 Department of Surgery, Tokyo Metropolitan Cancer and Infectious Diseases Center Komagome Hospital, Tokyo, Japan; 2 Department of Clinical Genetics, Tokyo Metropolitan Cancer and Infectious Diseases Center Komagome Hospital, Tokyo, Japan; 3 Hereditary Tumor Research Project, Tokyo Metropolitan Cancer and Infectious Diseases Center Komagome Hospital, Tokyo, Japan; 4 Department of Pathology, Tokyo Metropolitan Cancer and Infectious Diseases Center Komagome Hospital, Tokyo, Japan; 5 Department of Gastroenterology, Tokyo Metropolitan Cancer and Infectious Diseases Center Komagome Hospital, Tokyo, Japan; Okayama Daigaku, JAPAN

## Abstract

**Background:**

Lynch syndrome (LS) patients have a high risk of developing various tumors. This study aimed to clarify the characteristics of tumors developing in LS patients.

**Methods:**

This is a retrospective review of 55 LS patients treated at Tokyo Metropolitan Cancer and Infectious Diseases Center Komagome Hospital.

**Results:**

The median age at the diagnosis of the first malignant tumor and first LS-related tumor was 44 (range, 19−65) and 44 (range, 24−66) years, respectively. Of the 55 LS patients with developing malignant tumors, 45 (93.8%) developed an LS-related tumor as the first malignant tumor. Colorectal cancer (CRC) developed in 47 patients (85.4%), followed by endometrial cancer (n = 13, 56.5%) in females and gastric cancer (n = 10, 18.1%). In 6 gastric cancer patients, *Helicobacter pylori* was detected in resected specimens. Twenty-nine patients (52.7%) developed CRC and extra-colonic tumors; of these, 15 patients (48.3%) had mutations in *MLH1*, 10 (58.8%) in *MSH2*, and 4 (57.1%) in *MSH6*. At the age of 50, the cumulative incidence was 50.9% [95% confidence interval (CI), 36.9−63.3%] for CRC, 17.4% (95% CI, 5.2−35.6%) for endometrial cancer, and 5.5% (95% CI, 1.4−13.8%) for gastric cancer. Eight gastric cancer, one breast cancer patient, five bladder cancer patients, and one prostate cancer patient demonstrated loss of expression of the mismatch repair (MMR) protein; patients with thyroid cancer, spindle cell sarcoma, and giant cell tumors did not demonstrate this.

**Conclusion:**

Gastric cancer incidence was high in Japanese patients with LS and associated with *H*. *pylori* infection. MMR protein deficiency caused the development of malignant tumors in LS patients.

## Introduction

Lynch syndrome (LS) is an autosomal dominant disorder caused by germline mutations in one of the mismatch repair (MMR) genes, including the *MLH1* [1], *MSH2* [2], and *MSH6* genes [3]. Inactivation of MMR genes by germline and somatic mutations leads to a high frequency of replication errors in microsatellite regions and repetitive sequences in the coding regions of various growth-related target genes [4], resulting in the development of various tumors. According to Amsterdam criteria II [5], LS-related tumors are colorectal, endometrial, small bowel, and ureter/renal pelvis cancers. In the Revised Bethesda Guidelines, the following tumors are also included as LS-related tumors: stomach, ovarian, pancreas, biliary tract, brain (usually glioblastoma) tumors, sebaceous gland adenomas, and keratoacanthomas [6].

Most recent data concerning tumor development in LS patients have been reported from Western countries [7–13]; however, the details of tumors developing in LS patients from Asia have not yet been elucidated. Therefore, this study aimed to clarify the characteristics of tumors developing in Japanese patients with LS.

## Methods

### Patients

This study was approved by ethics committee of Tokyo Metropolitan Cancer and Infectious Diseases Center Komagome Hospital. All patients have given written informed consents. Fifty-five LS patients were selected from 34 LS families. Patients in whom “pathogenic”/“likely pathogenic” germline mutations of MMR genes were not detected were excluded.

Clinical information, including sex, date of birth, occurrence of tumors, date at the diagnosis of tumors, and *Helicobacter pylori* infection, was collected either from medical records or directly from patients.

According to the Revised Bethesda Guidelines, colorectal, endometrial, gastric, ovarian, pancreatic, ureter/renal pelvis, biliary tract, brain tumors, sebaceous gland adenomas, keratoacanthomas, and small bowel carcinomas were defined as LS-related tumors and other tumors were defined as non-LS-related tumors.

### Immunohistochemistry

For immunohistochemical staining, the following primary antibodies were used: Purified Mouse Anti-*MLH1* Monoclonal Antibody, clone G168-15 (BD Pharmingen, San Diego, CA) for *MLH1*; Anti-*MSH2* Antibody, clone FE11 (Calbiochem, La Jolla, CA) for *MSH2*; Purified Mouse Anti-*MSH6*, clone 44/*MSH6* (BD Pharmingen) for *MSH6*; and Purified Mouse Anti-*PMS2*, clone A16-4 (BD Pharmingen) for *PMS2*. Dilution rates of 50×, 50×, 100×, and 50×, respectively, were used. Staining was conducted using the DAKO EnVisionTM system (Agilent Technologies, Dako, Glostrup, Denmark), and diaminobenzidine (Sigma, St. Louis, MO) was used as the substrate chromogen. Normal colonic mucosa was used as the positive control.

### Statistical analysis

Data are presented as total, median (range), mean (95% confidence interval), or percentage (95% confidence interval). Statistical analyses were performed using Fisher’s exact test and the Mann–Whitney *U*-test. Cumulative cancer risks were calculated using the Kaplan–Meier method, and to compare risks between the two groups, the log-rank test was used. *P* < 0.05 was considered statistically significant. All statistical analyses were performed with EZR (Saitama Medical Center, Jichi Medical University, Saitama, Japan; http://www.jichi.ac.jp/saitama-sct/SaitamaHP.files/statmedEN.html), a graphical user interface for R version 3.4.1 (The R Foundation for Statistical Computing, Vienna, Austria) [14]. This interface is a modified version of R Commander version 2.4–0, which was designed to add statistical functions frequently used in biostatistics.

## Results

Table 1 shows the incidence of developed tumors in the 55 LS patients. Causative MMR genes were as follows: *MLH1* in 31 patients (56.3%), *MSH2* in 17 (30.9%), and *MSH6* in 7 (12.8%). Of the 55 these, 29 (52.7%) were females and 26 (47.3%) were males. The sex ratio was not significantly different across genes. In 45 (93.8%) of 48 LS patients with developing malignant tumors, LS-related tumors developed as the first malignant tumor.

**Table 1:** Characteristics in LS patients.

	All cases	*MLH1*	*MSH2*	*MSH6*
Number of patients	55	31	17	7
Gender (Female: Male)	29: 26	16: 15	8: 9	5: 2
All tumor	48 (87.3%)			
Colorectal	47 (85.4%)	26 (83.8%)	17 (100%)	4 (57.1%)
Endometrial **	13 (56.5%)	6 (42.8%)	3 (75%)	4 (80%)
Gastric	10 (18.1%)	7 (22.5%)	3 (17.6%)	0
Small bowel	4 (7.2%)	3 (9.6%)	1 (5.8%)	0
Renal/urinary tract	6 (10.9%)	2 (6.4%)	4 (23.5%)	0
Biliary	2 (3.6%)	2 (6.4%)	0	0
Pancreas	1 (1.8%)	1 (3.2%)	0	0
Ovary***	2 (11.7%)	0	1 (20.0%)	1 (50.0%)
Brain	1 (1.8%)	0	1 (5.8%)	0
Bladder*	6 (10.9%)	2 (6.4%)	4 (23.5%)	0
Breast (only female)*	4 (13.7%)	3 (18.7%)	1 (12.5%)	0
Cervix*	1 (3.4%)	1 (6.2%)	0	0
Thyroid*	1 (1.8%)	1 (3.2%)	0	0
Prostate*	1 (3.8%)	1 (6.6%)	0	0
Skin*	1 (1.5%)	0	1(4%)	0
Lymphoma*	1 (1.5%)	0	1 (4%)	0
Sarcoma*	1 (1.5%)	1 (3.2%)	0	0
Bone tumor*	1 (1.5%)	1 (3.2%)	0	0

*, non LS-related tumor

**, excluded patients who have had hysterectomy without malignancy

***, excluded patients who have had oophorectomy without malignancy

Colorectal cancer was the most common malignant tumor (*n* = 47, 85.4%), followed by endometrial cancer (*n* = 13, 56.5%) and gastric cancer (*n* = 10, 18.1%). Of the 47 colorectal cancer patients, 30 (63.8%) developed two or more colorectal tumors; the causative gene was *MLH1* in 15 patients, *MSH2* in 13 patients, and *MSH6* in 2 patients. Twenty-nine patients (52.7%) developed colorectal cancer and extra-colonic tumors; the causative gene was *MLH1* in 15 patients (48.3%), *MSH2* in 10 patients (58.8%), and *MSH6* in 4 patients (57.1%). Six patients had undergone hysterectomy without any gynecological malignancy. Excluding those 6 patients, 13 endometrial cancer patients (56.5%) were identified in total; the causative gene was *MLH1* in 6 patients (42.8%), *MSH2* in 3 patients (75%), and *MSH6* in 4 patients (80%). Breast cancer developed in 4 of the 29 females (13.7%). Of the 10 gastric cancer patients, all 6 treated in our hospital showed *Helicobacter pylori* in resected specimens.

Table 2 shows the first organ developing a malignant tumor for each gene. Colorectal cancer developed in 38% of the females and 85% of the males (*P* = 0.0007). Endometrial cancer was the second most common in female patients (31%).

**Table 2:** The first organ developed malignant tumor.

	Male				Female						
Tumor	Colorectal	Gastric	Ureter	None	Colorectal	Endometrial	Ovary	Bladder	Bone*	Cervix	None
*MLH1*	15	0	0	0	6	4	0	0	1	1	4
*MSH2*	7	1	1	0	4	2	1	1	0	0	0
*MSH6*	0	0	0	2	1	3	0	0	0	0	1
Total	22	1	1	2	11	9	1	1	1	1	5

*, non LS-related tumor

The median age at the diagnosis of malignant tumors is shown in Table 3. The median age at the diagnosis of the first malignant tumor and the first LS-related tumor was 44 (range: 19−65) and 44 (range: 26−66) years, respectively. There were no significant differences between females and males regarding the age at which the first malignant tumor and the first LS-related tumor developed.

**Table 3:** Median age at diagnosis of malignant tumors.

Cancer type	Median age at diagnosis (range)		
	All, year	Female, year	Male, year
The first malignant tumor	44 (19–66)	46 (19–63)	43 (26–66)
The first LS-related tumor	44 (26–66)	47 (29–63)	43 (26–66)
Colorectal	46 (26–74)	53 (29–69)	43 (26–74)
Endometrial	50 (40–57)	50 (40–57)	−
Gastric	62 (44–77)	65 (60–77)	47 (44–66)
Small intestine	62 (40–67)	64 (60–67)	51 (40–63)
Renal/urinary tract	58 (49–81)	64 (56–81)	51 (49–53)
Biliary	63 (55–72)	63 (55–72)	−
Pancreas	76	76	−
Ovary	43 (36–50)	43 (36–50)	−
Brain	49	−	49
Bladder*	54 (50–82)	59 (50−82)	54 (53–55)
Breast*	66 (44–78)	66 (44–78)	−
Cervix*	68	68	−
Thyroid*	65	65	−
Prostate*	74	−	74
Skin*	49	−	49
Lymphoma*	89	89	―
Sarcoma*	80	80	―
Bone cancer*	19	19	―

*, non LS-related tumor according to the Bethesda Guideline.

**Fig 1:**
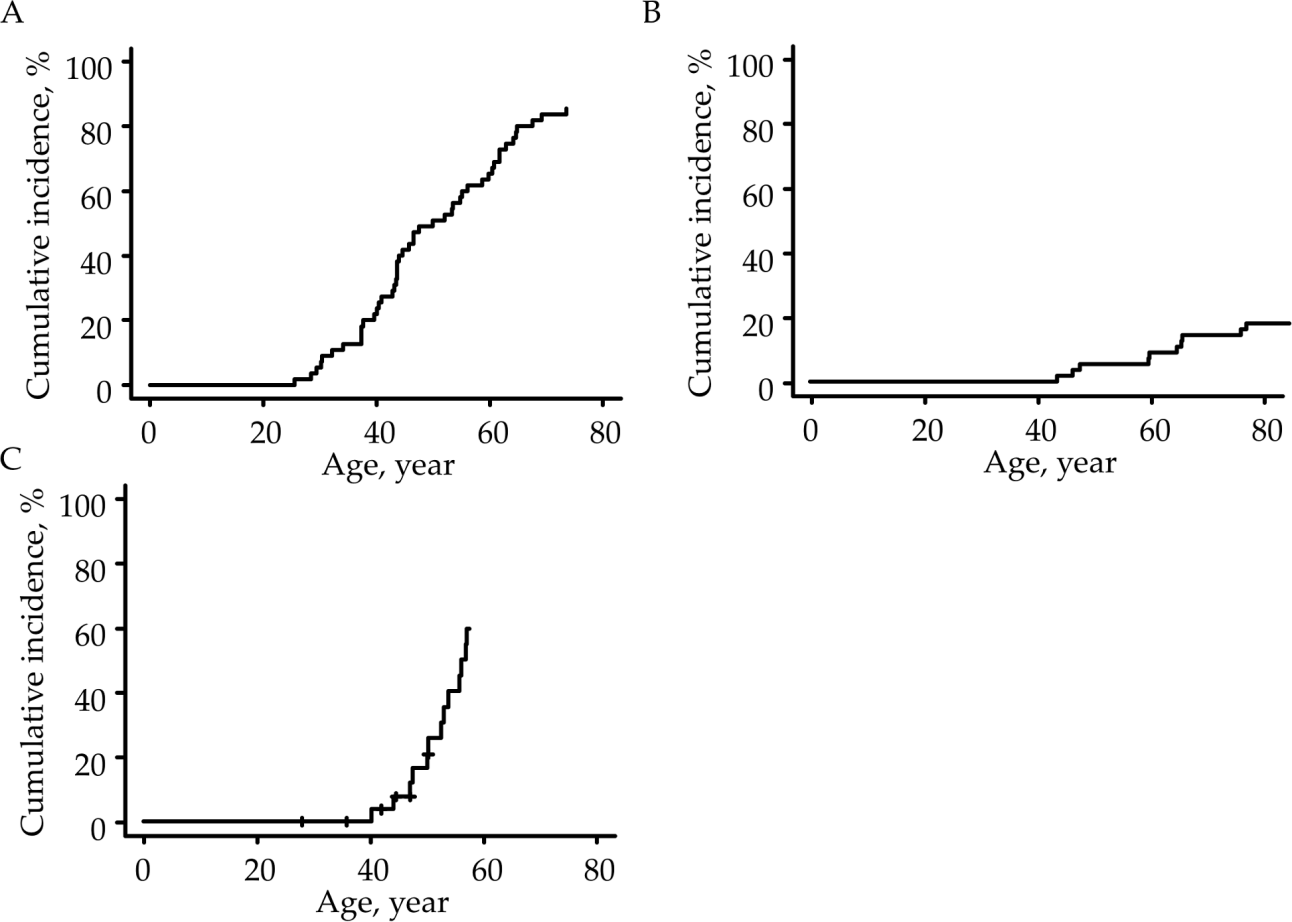
The cumulative incidence of malignant tumors in Lynch syndrome patients. (A) colorectal cancer, (B) endometrial cancer and (C) gastric cancer.

**Fig 2:**
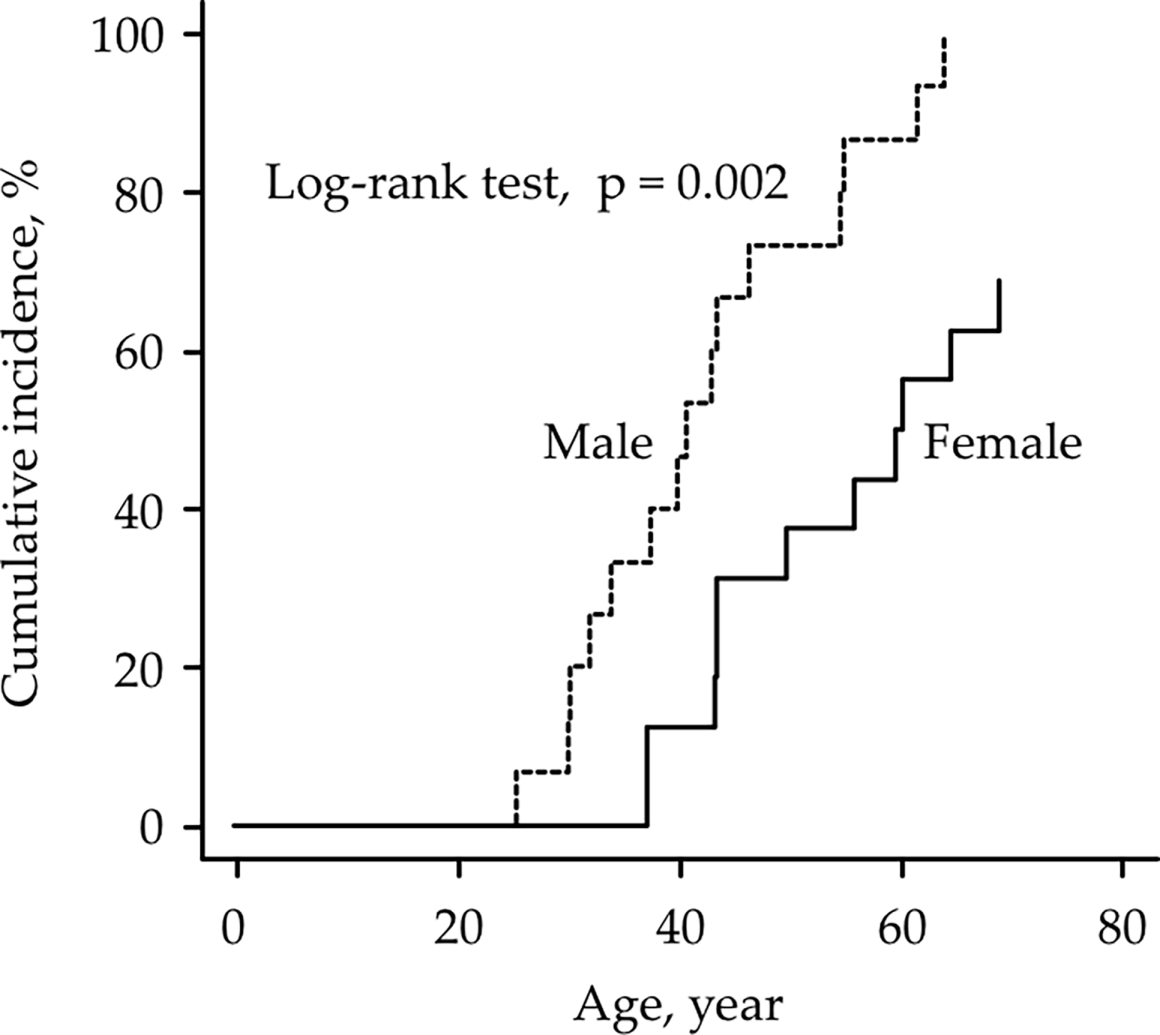
The cumulative colorectal cancer incidence in Lynch syndrome patients by gender.

Table 4 shows the age-specific cumulative incidence of colorectal cancer, endometrial cancer, gastric cancer, small intestinal cancer, and renal/urinary tract cancer in LS patients. At the age of 50, the cumulative incidence was 50.9% (Fig 1A) for colorectal cancer, 17.4% (Fig 1B) for endometrial cancer, and 5.5% (Fig 1C) for gastric cancer. The cumulative colorectal cancer incidence tended to be higher in males than in females across all eligible patients (*P* = 0.054). The cumulative colorectal cancer incidence in males was significantly higher than that in females in *MLH1*-mutated patients (*P* = 0.02) (Fig 2), while there were no significant differences in *MSH2*- and *MSH6*-mutated LS patients (P = 0.70 and P = 1.00, respectively).

**Table 4:** Age-specific cumulative incidence rates in Lynch syndrome patients.

			*MLH1*			*MSH2*			*MSH6*		
Cancer type	Age, year	All, %	Female	Male	p-value	Female	Male	p-value	Female	Male	p-value
Colorectal	50	50.9	37.5	73.3	0.029	50.0	77.8	0.700	0.0	0.0	1.000
	60	65.5	50.0	86.7		100.0	88.9		60.0	0.0	
	70	83.6	68.8	100.0		100.0	100.0		80.0	0.0	
Endometrial*	50	17.4	37.5	−	−	50.0	−	−	20.0	−	−
	60	56.5	50.0	−		50.0	−		60.0	−	
	70	56.5	68.8	−		87.5	−		80.0	−	
Gastric	50	5.5	0.0	20.0	0.522	0.0	0.0	0.498	0.0	0.0	−
	60	9.1	6.2	20.0		12.5	0.0		0.0	0.0	
	70	14.5	12.5	20.0		25.0	11.1		0.0	0.0	
Small intestine	50	1.8	0.0	0.0	0.603	0.0	1.1	0.346	0.0	0.0	−
	60	1.8	6.2	0.0		0.0	1.1		0.0	0.0	
	70	7.3	12.5	6.7		0.0	1.1		0.0	0.0	
Renal/urinary tract	50	1.8	0.0	0.0	0.945	0.0	1.1	0.542	0.0	0.0	−
	60	5.5	6.2	6.7		0.0	1.1		0.0	0.0	
	70	6.9	6.2	6.7		2.5	1.1		0.0	0.0	

*, excluded patients who have had hysterectomy without malignancy

**Fig 3:**
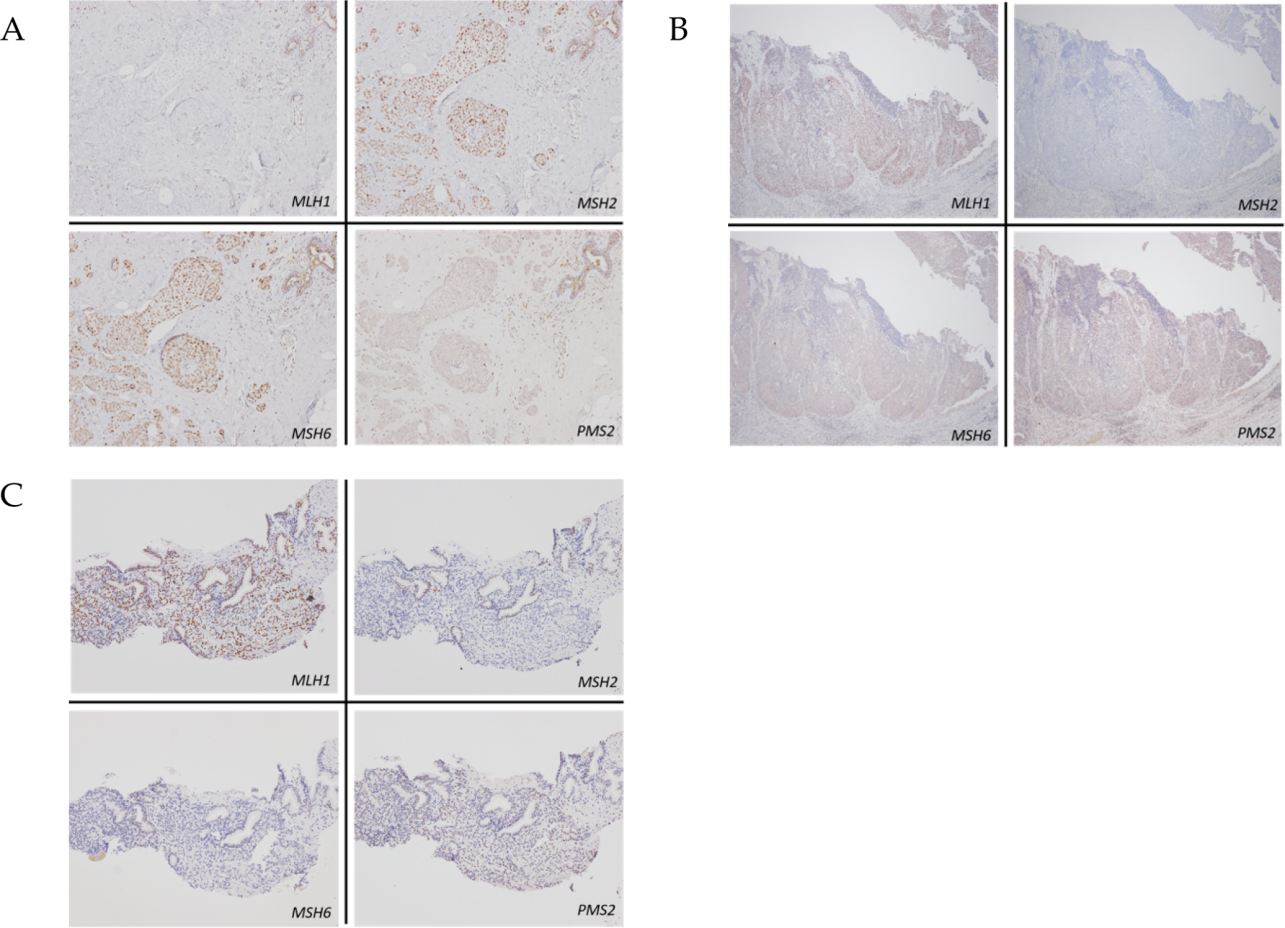
Immunohistochemical stainings for mismatch repair protein in malignant tumors in Lynch syndrome patients. (A) breast cancer with loss of *MLH1* and *PMS2* expressions, (B) bladder cancer with loss of *MSH2* and *MSH6* expressions and (C) prostate cancer with loss of *MSH2* and *MSH6* expressions.

In Table 5, the results of immunohistochemical staining for gastric cancer and non-LS-related tumors are presented. In gastric cancer, all 8 patients we treated in our hospital demonstrated loss of expressions of the MMR proteins. We treated 3 of 4 breast cancer patients, one of whom demonstrated a loss of expression of the MMR protein (Fig 3A). Additionally, 5 bladder cancer patients (Fig 3B) and 1 prostate cancer patient (Fig 3C) demonstrated loss of expression of the MMR protein. However, patients with thyroid cancer, spindle cell sarcoma, and giant cell tumors did not demonstrate a loss of expression of the MMR protein.

**Table 5:** The immunohistochemistry stains for gastric cancer and non-Lynch syndrome-related tumors.

Causative gene	Tumor	Immunohistochemistry steins			
		MLH1	MSH2	MSH6	PMS2
*MLH1*	Gastric cancer	-	+	+	-
*MLH1*	Gastric cancer	-	+	+	-
*MLH1*	Gastric cancer	-	+	+	-
*MLH1*	Gastric cancer	-	+	+	-
*MLH1*	Gastric cancer	-	+	+	-
*MLH1*	Gastric cancer	NA	NA	NA	NA
*MLH1*	Gastric cancer	NA	NA	NA	NA
*MSH2*	Gastric cancer	+	-	-	+
*MSH2*	Gastric cancer	+	-	-	+
*MSH2*	Gastric cancer	+	-	-	+
*MLH1*	Breast cancer	+	+	+	+
*MLH1*	Breast cancer	+	+	+	+
*MLH1*	Breast cancer	-	+	+	-
*MSH2*	Breast cancer	NA	NA	NA	NA
*MLH1*	Bladder cancer	-	+	+	-
*MSH2*	Bladder cancer	+	-	-	+
*MLH1*	Bladder cancer	-	+	+	-
*MSH2*	Bladder cancer	+	-	-	+
*MSH2*	Bladder cancer	+	-	-	+
*MSH2*	Bladder cancer	NA	NA	NA	NA
*MSH2*	Prostate cancer	+	-	-	+
*MLH1*	Spindle cell sarcoma	+	+	+	+
*MLH1*	Giant-cell tumor	+	+	+	+
*MLH1*	Cervical cancer	NA	NA	NA	NA
*MLH1*	Thyroid cancer	+	+	+	+
*MSH2*	Lymphoma	NA	NA	NA	NA
*MSH2*	Skin cancer	NA	NA	NA	NA

+, positive; -, negative; NA, not available because of treating in another hospital

## Discussion

In this study, we demonstrated tumor development in Japanese LS patients. These patients developed not only LS-related tumors but also non-LS-related tumors. In previous reports, the cumulative incidence at the age of 70 years was 54−74% for colorectal cancer, 28−60% for endometrial cancer, 5.8−13% for gastric cancer, 6.1−13.5% for ovarian cancer, 2.5−4.3% for small bowel cancer, 1.4−2.0% for biliary tract cancer, 0.4−3.7% for pancreatic cancer, 3.2−8.4% for ureter/renal pelvic cancer, and 2.1−3.7% for brain cancer [7–13]. Cancer risk has been reported to be different among MMR gene mutation carriers [15, 16]. The current study demonstrated the risk of various tumors, such as colorectal, endometrial, gastric, small bowel, and ureter/urinal pelvic cancer, in LS patients. In all tumors except gastric cancer, tumor risks were similar to those reported in past reports [11, 12, 17]. However, the cumulative incidence of gastric cancer was higher in Japan than in Western countries, which was similar to the result in a previous report from Japan [18]. In East Asia, including Japan, gastric cancer is common in LS patients [18, 19]. It has been proposed that the development of gastric cancer is associated with *H*. *pylori* infection [20]. which is common in Asia [21]. In the present study, all 8 LS patients with gastric cancer we treated had *H*. *pylori* infection and also had loss of expressions of the MMR proteins. Thus, these findings support the proposal that *H*. *pylori* infection increases the risk of gastric cancer in LS patients.

In the present study, we found non-LS-related tumors in LS patients: 6 patients with urinary bladder cancer, 4 with breast cancer, and 1 patient each with cervical cancer, thyroid cancer, prostate cancer, skin cancer, lymphoma, sarcoma, and bone cancer.

Recent reports have proposed that breast, urinary bladder [22]. and prostate [23] cancers are also LS-related tumors. In the present study, we detected loss of expression of the MMR protein in 1 breast cancer patient, 5 urinary bladder cancer patients, and 1 prostate cancer patient. A report on breast cancer in LS patients by Lotsari *et al*. demonstrated that 65% of breast cancer tissues showed reduced or no MMR protein expression, corresponding to the germline mutation [24]. Gylling *et al*. reported that all 4 urinary bladder cancer patients with LS showed decreased MMR protein expression [25]. Moreover, a recent report has indicated that 70% of prostate cancer patients with LS demonstrated loss of expression of respective MMR proteins [26]. Therefore, these cancers are considered to be LS-related tumors in LS patients.

However, it is controversial whether thyroid cancer is an LS-related tumor. There are only few case report concerning thyroid cancer in LS [27–29]. The age-adjusted incidence of thyroid cancer was 21.0 per 100,000 women in the United States and 12.3 per 100,000 women in Japan, and the lifetime risk of developing thyroid cancer in women was 1.79% in the United States and 1.26% in Japan [30, 31]. In the present study, thyroid cancer patients did not show a loss of expression of the MMR protein. Thus, it is difficult to say that the incidence of thyroid cancer is high in LS patients because thyroid cancer developed in only 1 of the 55 LS patients.

The present study has the following limitations: (1) selection bias was caused by the retrospective nature, (2) treatment data were lacking, and (3) it was a single-center study. Nonetheless, considering that there are only a few published studies on LS-related cancers in Asia, we believe that our findings will help researchers and physicians clarify the nature of LS. However, further studies are required to overcome these limitations.

In conclusion, gastric cancer had a high incidence in Japanese patients with LS and was associated with *H*. *pylori* infection. MMR protein deficiency causes malignant tumors to develop in breast, urinary bladder, and prostate tissues in LS patients.
